# OLYMPUS-POPGEN: A synthetic population generation model to represent urban populations for assessing exposure to air quality

**DOI:** 10.1371/journal.pone.0299383

**Published:** 2024-03-08

**Authors:** Arthur Elessa Etuman, Taos Benoussaïd, Hélène Charreire, Isabelle Coll

**Affiliations:** 1 AME-SPLOTT, IFSTTAR, Univ Gustave Eiffel, Marne-la-Vallée, France; 2 CNRS, LISA, Université Paris Est Créteil et Université Paris Cité, Créteil, France; 3 Lab-Urba, Département de Géographie, Institut d’urbanisme de Paris, Université Paris-Est Créteil, Paris, France; Addis Ababa University, ETHIOPIA

## Abstract

**Scientific question:**

With the new individual- and activity-based approaches to simulating exposure to air pollutants, exposure models must now provide synthetic populations that realistically reflect the demographic profiles of individuals in an urban territory. Demographic profiles condition the behavior of individuals in urban space (activities, mobility) and determine the resulting risks of exposure and environmental inequalities. In this context, there is a strong need to determine the relevance of the population modeling methods to reproduce the combinations of socio-demographic parameters in a population from the existing databases. The difficulty of accessing complete, high-resolution databases indeed proves to be very limiting for the ambitions of the different approaches.

**Objective:**

This work proposes to evaluate the potential of a statistical approach for the numerical modeling of synthetic populations, at the scale of dwellings and including the representation of coherent socio-demographic profiles. The approach is based on and validated against the existing open databases. The ambition is to be able to build upon such synthetic populations to produce a comprehensive assessment of the risk of environmental exposure that can be cross-referenced with lifestyles, indicators of social, professional or demographic category, and even health vulnerability data.

**Method:**

The approach implemented here is based on the use of conditional probabilities to model the socio-demographic properties of individuals, via the deployment of a Monte Carlo Markov Chain (MCMC) simulation. Households are assigned to housing according to income and house price classes. The resulting population generation model was tested in the Paris region (Ile de France) for the year 2010, and applied to a population of almost 12 million individuals. The approach is based on the use of census and survey databases.

**Results:**

Validation, carried out by comparison with regional census data, shows that the model accurately reproduces the demographic attributes of individuals (age, gender, professional category, income) as well as their combination, at both regional and sub-municipal levels. Notably, population distribution at the scale of the model buildings remains consistent with observed data patterns.

**Conclusions and relevance:**

The outcomes of this work demonstrate the ability of our approach to create, from public data, a coherent synthetic population with broad socio-demographic profiles. They give confidence for the use of this approach in an activity-based air quality exposure study, and thus for exploring the interrelations between social determinants and environmental risks. The non-specific nature of this work allows us to consider its extension to broader demographic profiles, including health indicators, and to different study regions.

## 1. Introduction

Human activities have significant impacts on our environment and populations, particularly through air, water and soil pollution. In urban areas, chronic exposure to atmospheric pollutants can result in severe respiratory and cardiovascular problems throughout life [1–3]. The distribution of these environmental nuisances among individuals and regions exhibits substantial disparities, leading to the issue of environmental inequalities [4]. Given the social implications of these issues, it is imperative to design methods for identifying and characterizing these inequalities, with a view to devising solutions to reduce or mitigate their negative impacts.

The quantification of exposure to environmental nuisances in urban areas is still mostly done by cross-referencing census data and/or surveys with spatialized environmental data. Yet, the issue of exposure to atmospheric pollutants goes well beyond the living area. The literature has acknowledged the importance of individuals’ socio-demographic characteristics as drivers for their diurnal mobility and energy consumption behaviors, which in turn determine the intensity of pollutant emissions, and ultimately shape the daily exposure of individuals along their activities and trips in the city [5]. Therefore, the priority is to develop new environmental risk models based on the representation of the relationships between individuals, behaviors, activities, and exposure to air pollutants.

To be relevant, these models need to be based on a realistic representation of populations with the social and demographic properties that impact their daily activities and mobility. However, databases on the characteristics of urban populations may be incomplete or lack spatial resolution, but above all for reasons of confidentiality or plurality of sources, they cannot meet the need to define individuals with their own complete and coherent social profile. Synthetic populations are simulated data sets that reproduce the demographic, socio-economic, and behavioral characteristics of a real population and as so, they are a suitable solution for overcoming these limitations [6]. Based on a defined set of parameters such as age, gender, professional activity, household composition and income, they are recognized for being statistically representative of the population, although they don’t reproduce the characteristics of every individual [7]. Synthetic populations are generally created using resampling and matching techniques, allowing access to statistically representative data without violating individuals’ confidentiality [8]. In the end, they represent an added value by enabling more detailed exposure analyses [9].

Synthetic population simulation involves micro-simulation [10], an approach which consists of reproducing individual behaviors observed in surveys or censuses, on representative samples of the populations studied. Microsimulation can be combined with multi-agent systems (MAS) that can capture and simulate interactions between individuals (agents) and between agents and their environment. MAS can be applied in various domains, including transportation modeling [11–13], environmental studies [14], and health studies [15]. Over the last ten years, we have seen the emergence of work cross-referencing MAS with environmental data, providing more realistic exposure data for a larger number of individuals than is possible with sensor measurements [16–18].

The landscape of synthetic population modelling covers a wide range of microsimulation approaches. Their success often depends on the fitting process of the demographic data towards aggregated data, which is a significant determinant of the model’s final form [6, 19–22]. In this frame, the works of [23] highlighted some advantages of the Markov Chain Monte Carlo (MCMC) approaches over more conventional methods based on iterative proportional fitting (IPF). MCMC methods are computer simulation techniques for random sampling from probability distributions [24]. Since then, they have become widely recognized as a standard in synthetic population modeling [25].

As will be discussed in the methodology section, we have chosen a MCMC method to produce a population intended to feed our environmental exposure risk model. The innovative aspect of our work is linked to our ambition to integrate population characteristics into a platform for modeling the risk of exposure to pollutants. First, this requires placing specific emphasis on the production of coherent socio-demographic profiles of individuals and households, and not just on the distribution of parameters by neighborhood. Indeed, combinations of individual parameters and their links to household can be used to predict demographically and socially differentiated mobility and energy consumption behaviors—which will in turn lead to differentiation in the associated environmental risks. In literature, while this issue has recently been addressed by certain authors (e.g., [25–27]), it is not yet a standard on the subject. Most authors work either at household level [28] or individual level [29, 30], without looking at/analyzing the consistency between those two levels. [23] indicates that this is a perspective of interest. Moreover, our work further explores the socio-differentiation of individuals in the synthetic population, by associating households with dwellings according to their income with the idea of addressing inequalities in exposure to road nuisances.

The aim of this paper is to provide an assessment of the ability of our approach to produce a synthetic population with coherent combinations of socio-demographic profiles of individuals at different levels and administrative scales, and to evaluate its relevance to an increasing diversity of parameters.

The paper proceeds as follows. The OLYMPUS-POPGEN approach and set-up section discusses the choice of a general approach for OLYMPUS-POPGEN, and the data used for its application over the Paris region. The OLYMPUS-POPGEN operational processing section describes the code structure and principles, detailing the mechanism of the generation of the population from probability distributions, and using conditionality. It also illustrates the application of this methodology over the Paris area. Before concluding, The Results and discussion section presents the results of the population modeling process as well as their evaluation. The conclusion summarizes our main findings and their implications, as well as future research needs. Throughout the paper, we highlight the potential of this approach to use supplementary data to validate our approach for further research, which will include health and vulnerability indicators in the diagnosis of environmental risks.

## 2. OLYMPUS-POPGEN approach and set-up

OLYMPUS-POPGEN is an adaptable Python package specifically designed for the generation of synthetic populations, households, and housing units. Under a GNU open-source license, this code includes a data preprocessor, a synthetic population generator, and a housing allocator. It forms an integral part of the OLYMPUS, an emission modeling platform which relies on an activity-based travel demand model to produce diurnal mobility matrices and modal share for all individuals of the synthetic population, over the region of interest [31]. Mobility matrices are used to produce OLYMPUS’s air pollutant emission inventory, which is further used in an air quality model. They also form the basis for calculating exposure throughout the day for all individuals in the city. In this paper, we will exclusively focus on the POPGEN part of the OLYMPUS platform.

### 2.1 General approach

Several approaches can be implemented to model synthetic populations depending on the quantity, type and quality of data available [32]. Each has its own strengths and limitations, and we have explored the methods available to identify the most suitable and promising for our ambitions.

[22] have proposed a classification that distinguishes methods in Synthetic Reconstruction (SR), Combinatorial Optimization (CO) and Statistical Learning Methods (SL). All these approaches are not exclusive and can be combined. Synthetic Reconstruction (SR) is the most used approach for building synthetic populations. It is quite demanding in terms of input data as it requires access to both samples and aggregated data, and fitting is made by applying weights to individual and household characteristics prior to replication. It includes methods like Iterative Proportional Fitting (IPF), Iterative Proportional Update (IPU), and Hierarchical Iterative Proportional Fitting (HIPF) [33]. Despite their effectiveness, these methods notably struggle with the "zero-cell problem," a problem of under-representation of certain attribute combinations, which worsens as attribute combinations multiply. This remains a major problem requiring unbiased solutions [34]. Furthermore, IPF methods structurally fail to restore the joint distribution of attributes at the household and individual levels [35]. Combinatorial Optimization (CO) is based on randomly selecting combinations of individuals and households to fit the marginal characteristics of each small geographical area. Fitting is improved in an iterative process by weighting, exchanging or adding new elements. Still, this term covers stochastic approaches that cannot explicitly determine the joint distribution of all controlled attributes. And although they can produce accurate populations, the numerical resolution of CO-based techniques is complex. Their application to large populations requires excessive computation times and may fail to find the optimal solution [22, 35]. In addition, the related approaches such as simulated annealing [36–38] often face the issue of over-representation of sample data due to repeated cloning [23, 39]. As such, these two groups of methods don’t seem to be the best suited to our constraints.

The third group of approaches is composed of statistical Learning (SL) methods which cover various techniques and algorithms, that learn the demographic and socioeconomic attributes of the real population to model them. They include approaches based on Markov processes, "fitness"-based synthesis [40], Bayesian networks [41], and copula-based methods [42]. What’s interesting for our purposes is that these methods focus directly on restoring the joint distribution of attributes in the sample, based on an estimate of the probability of possible attribute combinations. They are therefore able to compensate for the lack of heterogeneity in the input data, although they may have difficulty in representing some marginal combinations by establishing a conditional distribution. Due to their structure, they are less demanding in terms of volume and data sources and can be implemented using population samples only. While promising, it should be noted that SL approaches are also limited in terms of the computational time required, which makes their applications to large populations cumbersome.

Based on this analysis, we oriented our choice towards an SL approach, and more specifically towards a Markov Chain Monte Carlo (MCMC) method. MCMC methods are simulation algorithms that generate sequences of samples distributed according to a law of interest, starting with a random probability distribution and progressing progressively towards the desired probability distribution [43]. This approach is based on the production of a dependent sequence of random draws by stochastic processes. For the synthetic population, probability estimates for all parameter combinations are generated from partial views of these combinations in the sample. Thus, this approach can overcome the main drawbacks associated with fitting in previous approaches, and MCMC can reproduce better population heterogeneity with small samples. Known for its adaptability and its wide area of application, the MCMC method is indeed theoretically able to converge to the target distribution while preserving variable dependencies (in our case between the 2 levels “household” and “individual”). [23] have described in detail the implementation of such a method, using discrete choice models to construct conditional distributions of individuals’ attributes and using the Gibbs sampler as the MCMC algorithm after conditionals have been obtained. The synthetic population is then obtained by drawing the number of individuals/households corresponding to the required population size [23, 44]. This is the approach we have adopted to design the OLYMPUS-POPGEN model.

### 2.2 Description of the study area

**Fig 1:**
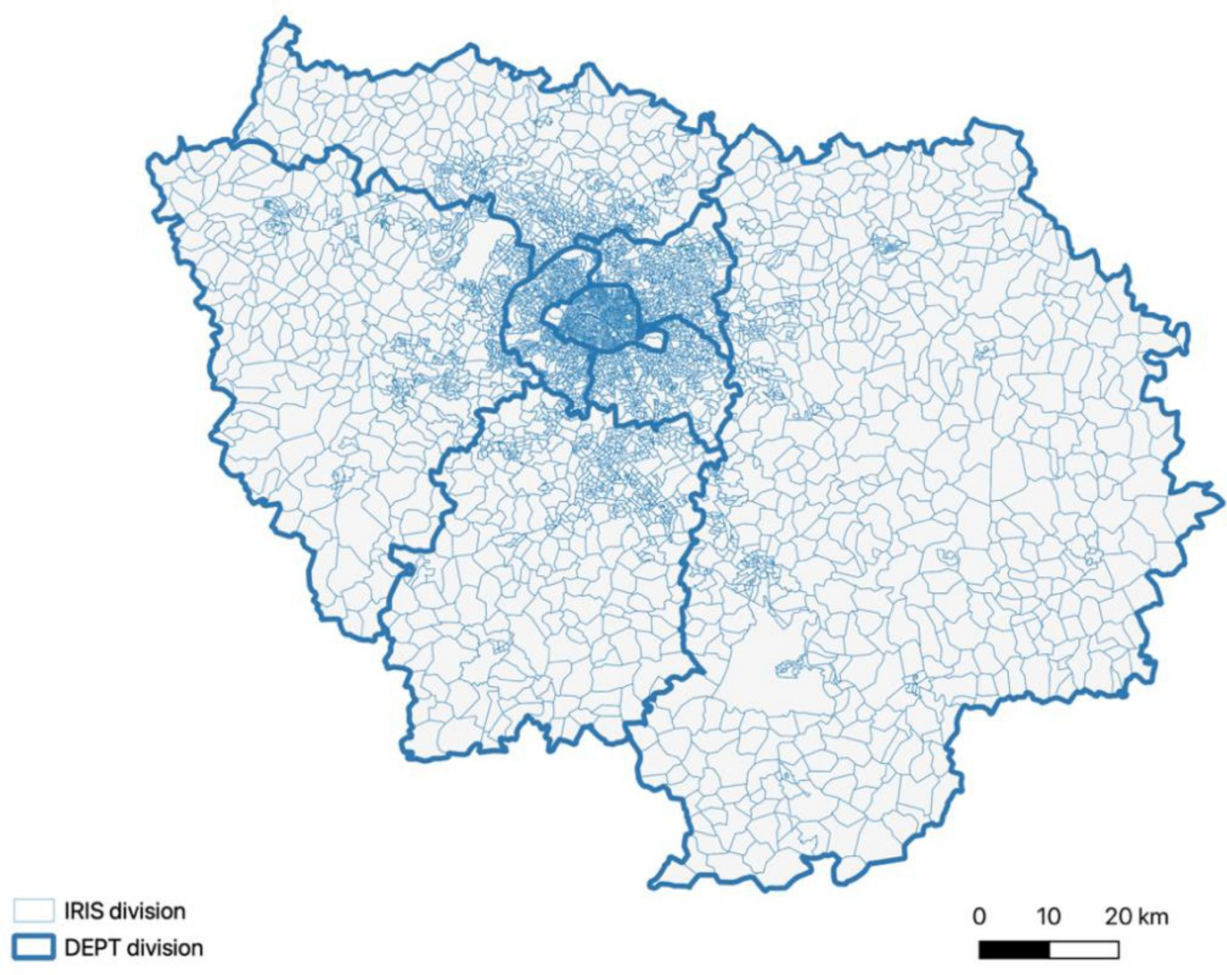
Map of the Île-de-France region displaying the eight departments and secondary administrative subdivisions comprising the 5200 IRIS units.

The area of study is the Île-de-France region, with Paris in its center. This region is subdivided into eight departments, 1281 municipalities or "communes," and 5259 infra-municipal statistical units or "IRIS"—the finest level of statistical analysis available. IRIS, which stands for “grouped areas for statistical information”, is a sub-municipal division based on geographical and statistical criteria with 1,000 to 5,000 inhabitants on the average, and which is as far as possible homogeneous in terms of housing. With a population of 11,786,234 in 2010, the year of simulation, the region accounts for approximately 18% of the total French population while occupying roughly 2% of the national territory. This disparity results in a densely populated area, characterized by significant social discrepancies [45]. Achieving sustainable urbanism and air quality are two of the region’s paramount environmental challenges. ***Fig 1*** illustrates the region along with its departments and IRIS units.

### 2.3 Input data

Data relating to territories is generally organized according to four levels of spatial divisions: a regional layer divided into departments; a sub-layer called "CANTON-VILLE," which covers electoral districts within departments; a following sub-layer comprising "COMMUNES"; and a final sub-layer at the IRIS level (as shown in ***Fig 1***).

The data used to force OLYMPUS-POPGEN primarily originates from the French National Institute of Statistics and Economic Studies (INSEE) population census (RGP) (for 2010), the French topographical database (BDTOPO), and the French localized social and fiscal files (FILOSOFI) (for 2010).

In this project, we used the census data available at the IRIS level as supplied by INSEE. RGP data, although not exhaustive, provides details on characteristics such as age, gender, household status, household type, and household size. The income database furnishes information on the median income of individuals and households at various geographical levels. This information is collected annually by INSEE at the household level throughout France. For the distribution of individual incomes, the relevant scale was the communal level.

We employed the Demand for Real Estate Value (DVF) database published and produced by the Directorate General of Public Finances, which delivers information on a building’s sale price for distributing households in homes according to their income.

We also integrated health data, particularly indicators of overweight and obesity. Individual level overweight and obesity data are derived from the Obepi2 survey, which provides 2020 data by sociodemographic profile (i.e., considering occupations and socio-professional categories (SPC), age, sex) for adults, on a national scale. This study has been a benchmark for obesity monitoring in France since 1997 (11,800 people were surveyed in the last study) and is the subject of numerous publications on the subject [46, 47]. To generate housing and distribute households, we utilized the BDTOPO database produced by the National Institute of Geographic and Forestry Information (IGN). This database provides information about buildings, their type, height, area, and the number of housing units per building.

## 3. OLYMPUS-POPGEN operational processing

In this section, we describe the structure and operational mechanisms of the OLYMPUS-POPGEN simulator for individuals, households and dwellings. Unlike traditional approaches where individuals are created disjointly and then redistributed within households by association, our approach co-generates individuals within the same household, like the process adopted by [44].

The generation of our synthetic population involves characterizing and spatializing demographic and socio-economic information based on a conditional probability distribution. The process of synthetic population modeling encompasses four main stages:

- Stage 1. Pre-processing of databases.
- Stage 2. Definition of control attributes.
- Stage 3. Definition of attributes of households and individuals of the households following an MCMC model.
- Stage 4. Generation of housing and allocation of households to a dwelling.

### 3.1 Stage 1: Pre-processing of databases

The first stage involves pre-processing the database. The pre-processing phase involves managing missing and unknown values, converting into required types, discretization in attribute selection, encoding categorical data, comparing, and adjusting various category attributes.

The first step is to identify the hierarchical structure of the data, i.e., the administrative groups into which they can be aggregated. They include levels such as departments, cantons, communes, and IRIS, depending on the type of data used. It is then critical to identify the explanatory variables to be included in the Bayesian hierarchical model, focusing on the specific characteristics of each level or on characteristics shared between levels.

Data cleaning is also a significant step to eliminate missing values, data entry errors, and outlier data. To fill the gaps of certain missing data—unavailable due to privacy reasons or too small a sample—when information is missing for certain variable combinations at the IRIS level, we use data from more aggregated spatial levels.

Every individual in a real population has a specific set of attributes, which will be represented in the OLYMPUS-POPGEN synthetic population by a vector X = {X1, X2,…, Xn}, where n represents the number of attributes to be synthesized. ***Table 1*** lists and describes the OLYMPUS-POPGEN variables and their classification, as they were used for the synthetic population generation. The utilized data are both qualitative (e.g., gender, age, income, education, employment) and deterministic quantitative (e.g., household size, car ownership, household type, household role, number of children, language). For this work, the minor occurrences of some variables were filtered to simplify the representation of households and individuals. All data sources are available in ***Table A1 in S1 Appendix***.

**Table 1:** Table of attributes. List of the main variables characterizing synthetic populations as well as their type and the categories created for their implementation in POPGEN.

Level	Variable	Type	Categories
Zonal	Population and density	Integer	
Household	Type	Categorical	Single, single parent, couple, family
	Household size (hh_i_)	Integer	[1,2,3,4,5,6]
	Income	Continuous	
	Car ownership	Integer	[0,1]
Individual	Age	Categorical	[0–15;15–35;35–65;65–120]
	Gender	Categorical	[Male; Female]
	SPC	Categorical	[1, 2, …, 8]
	Level of education	Categorical	none, primary, secondary, university/college
	Statut	Categorical	[head, spouse, child]
	Main activity	Categorical	[Employed, unemployed, retired, student, other unemployed].
	Overweight	Integer	[0,1,2]

### 3.2 Stage 2: The conditionals

In this step, the quality of the simulation results is determined by the quality of the control attributes. We defined multidimensional control variables to improve the accuracy of correlations between variables. Conditions were derived from census data for each statistical domain. Some variables, such as income or overweight, are independent of the other control variables as they rely on separated sources. The construction of these conditions is based on modelling assumptions to capture the main correlations in the database. We have chosen to model the household first (location, size and type) and then assign characteristics to each individual within it.

#### 3.2.1 Sociodemographic attributes

To avoid unrealistic situations and for the sake of simplicity, we restrict the construction of synthetic populations while preserving the correlations with a set of hypotheses. These apply notably to the status of the spouse in the family, the gender of the spouse and the minimum age difference between mother and child.

Thus, the status of the head of household is systematically associated with the first agent generated, and the status of the spouse is associated with the second agent, while the 3rd and subsequent individuals are always children of the household. The exceptions to this rule are "Single" household (one-person households automatically labelled as such) and "single-parent families". Once the gender of the head of household has been determined, the opposite gender is assigned to the spouse.

Also, some parameters related to age are restricted in the model. It concerns more specifically the age difference between the head of household, the mother and the children. One modelling assumption is that the head of household is at least 21 years old, with a minimum age limit of 18. Then, individuals under 18 are systematically schoolchildren with inactive SPC. The default SPC for people over 65 is "retired".

#### 3.2.2 Income attributes

The generation of income attributes relies on the professional categorization of individuals and on local statistics. The distribution of income values is based on the methodology used by the Mobisim modeling platform in [48], which enables to reproduce infra-communal variability based on the social characteristics of individuals. In this methodology, the commune’s median income is weighted by a coefficient that depends on an individual’s SPC to produce its income attribute. We have assumed that annual income cannot be less than the minimum wage (12,672 € in 2010) for a working person, and less than the Active Solidarity Income (5,520 € in 2010) for a non-working person aged 25 or over.

The weight is calculated as shown in (***Table A2 in S1 Appendix***) with α = coefficient per SPC, r_SPC_ = median annual income of the SPC and r = median income of the population. Median annual incomes by SPC are taken from the [49]. When α is greater than 1, this means that the median salary of the SPC will be higher than the average salary of the total population, and vice versa.

#### 3.2.3 Overweight and obesity attributes

The attribution of the overweight/obesity variable is based on national data from the Obepi survey which we transposed to our population in Île-de-France. This variable depends on the gender, age and SPC of each agent. It is then randomly distributed to agents in the associated sub-groups. The contingency table is defined by territory based on the total number occurrences. This is an exploratory methodology that needs to be refined, but its value lies in the fact that it is based on a robust distribution of socio-demographic properties associated with health problems.

### 3.3 Stage 3: MCMC-based synthetic population generation

**Fig 2:**
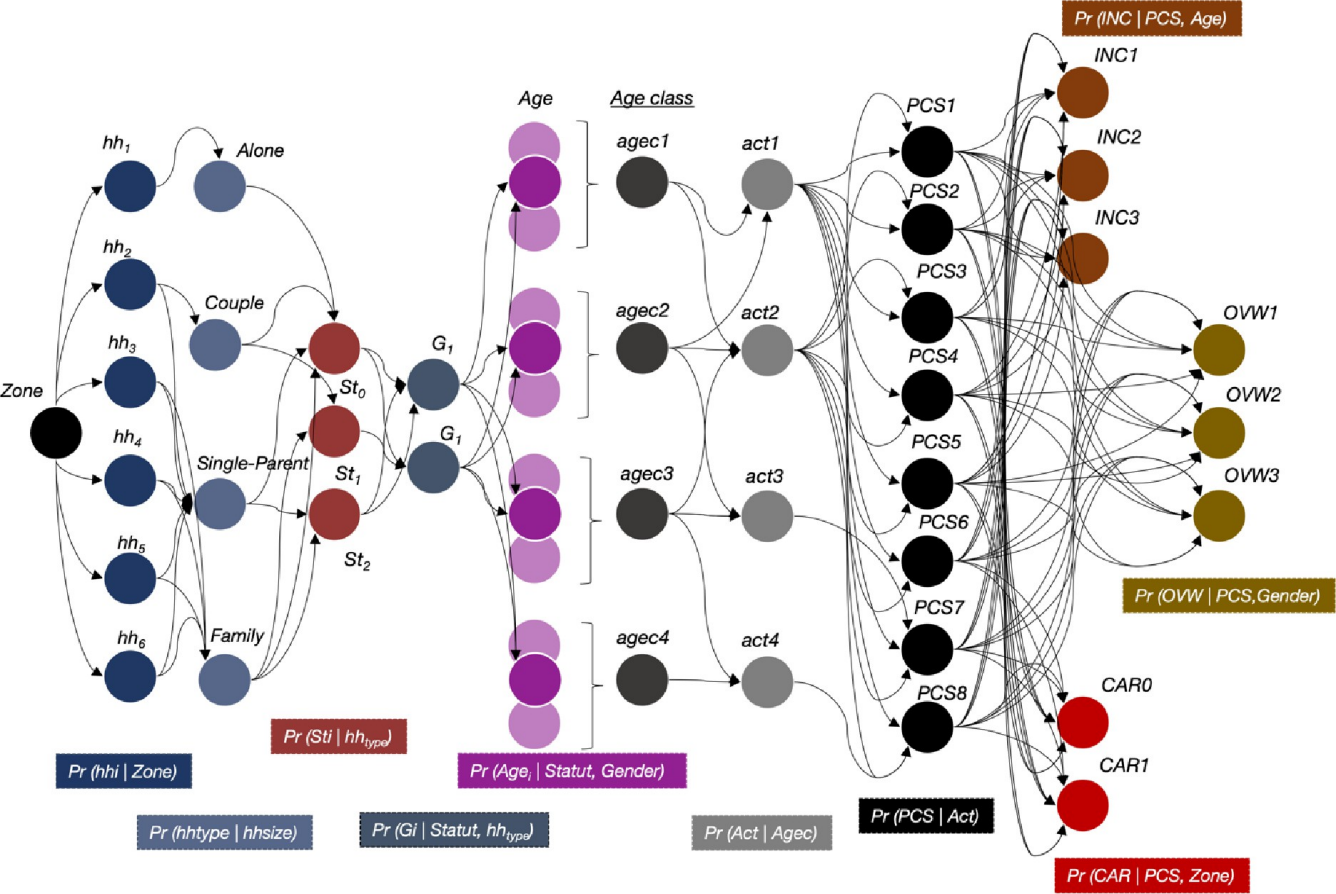
Diagram of the conditional probability tree–each circle represents a characteristic associated with a household and an individual in the household. Each geographical unit is associated with the probability of the number of individuals making up a household. This in turn determines the type of household and the position of the generated individual within the household. Age is then determined, and from this derives the other socio-demographic characteristics that characterize every individual.

In this step, we combine household size distribution fitting with the MCMC method we described above, which samples from complex probability distributions to provide a representative distribution of the observed parameters. We specifically use Gibbs sampling–a specific declination of the MCMC algorithm–that simulates the joint distribution of attributes by successively sampling each attribute conditionally on all the others. This method is particularly suitable for generating realistic populations as it preserves the dependencies between variables while avoiding the complex calculations associated with the joint distribution. In the Gibbs sampling algorithm, each parameter is updated by sampling from its full conditional distribution, while keeping the other parameters fixed at their current values. This is done iteratively. Each iteration involves going through each parameter and updating it using its full conditional distribution given the data and the current values of the other parameters. After many iterations, the samples drawn from these full conditional distributions will converge to samples from the joint posterior distribution of interest. Detailed equations can be found in [23]. The construction principle and the linkage between the different variables are described in the diagram in Fig 2.

In our methodology, the starting point for the simulation is the number of individuals located in the area under consideration (IRIS) (***Fig 2***, black bubble). Then, based on the distribution of household size in the area (derived from the training sample), the model establishes a set of households that are discretized according to their size. In the model, four types of households can be distinguished according to family structure (i.e., the number of people in the household) (***Fig 2***, dark blue bubbles). To ensure an accurate and realistic representation of the population in each IRIS, we run an optimization step between the total number of households, the total number of individuals and the household size distribution.

For each household, the model then generates the socio-demographic characteristics of the individuals, as previously defined by the X vector. This step is based on the MCMC method and Gibbs sampling. First, age is assigned according to household type and household status (***Fig 2***, purple bubbles), then a professional activity is assigned according to age (***Fig 2***, grey bubbles). These characteristics are then used to assign a socio-professional category (***Fig 2***, black bubbles).

### 3.4 Stage 4: Generation of housing and allocation of households to a dwelling

#### 3.4.1 Housing generation

To accurately locate the city’s agents and households at the dwelling level in blocks of flats or individual houses, we first need to determine the number of dwellings available in each IRIS). The starting point here is the BDTOPO building database. This database allows buildings to be filtered by type of use and provides information on the surface area of the building and its height, as well as the number of dwellings in the building. However, in some cases the information on the dwelling was missing, so we had to recreate it.

To do this, we based our method mainly on the type of building, the specific height and the floor area of the building. With regional estimates of the average size of a dwelling, we can define an average dwelling size for the case study, using data from the APUR report, 2017 [50], with approximately 59 m^2^ for the city center area, 69 m^2^ for the inner suburbs, and 89 m^2^ for the outer suburbs. Based on the characteristics of the building (height, width, surface area) and local land-use data, we predict whether it is a house, or a block of flats as well as the number of dwellings available based on the ratio between the total surface area of the building and the average size of the dwellings. Ultimately, ~5.2 million housing units are generated at the regional level, which stand for to the 5.1 million housing units existing in the region according to the 2013 housing survey.

To facilitate the allocation of housing to households based on income, we have categorized housing by price category. To do this, we cross-referenced the available data on house prices with the built-up areas of the BDTOPO to estimate the housing classes at the building scale. In this work, housing classes are estimated using a classification for 3 classes of housing type (low-intermediate-high).

#### 3.4.2 Allocation of households

Once the dwelling generation stage is complete, the dwellings are allocated to households according to the income bracket in which they fall, as far as possible.

The aim here is not to define the right number of dwellings in the area, but to allocate the location of households on a finer scale than the IRIS and to reproduce the spatial heterogeneity of the neighborhood. Households are allocated to dwellings based on their income and the estimated value of dwellings in the neighborhood in which they live. High-income households are randomly allocated to dwellings in the high-value category, then middle-income and low-income households are randomly allocated. If the stock of dwellings in a class is exhausted, the model assigns an associated dwelling to the nearest class. As an example, within our illustrative 40-unit building, if 15 units are of high valuation, 12 of intermediate worth and the remainder 13 of lower fiscal value, households with incomes in the upper decile are preferentially allocated to the high-valued units. As allocation progresses, should high-valued units reach saturation, subsequent allocations pivot to the middle- and lower-income strata.

## 4. Results and discussion

In this section we present the results of population modeling over the Paris region. The census provides the attributes of individuals and households for a total of just under a third of the total regional population, i.e., close to 4 million people.

There are several methods available for validating synthetic population models, which can be divided into two broad categories: endogenous validation and exogenous validation. Exogenous validation involves comparing simulated results with a data source external to the model. Endogenous validation is based on a single database, separated into a training set and an evaluation set. We have chosen the latter approach to evaluate the statistical properties of the modeled population. For the population synthesis, we first selected a subset representing approximately 66% of the data for model training and the remaining 33% for model evaluation ("testing"). In addition, we sampled the marginal distribution of each variable, generating its value independently of the rest of the variables.

The validation of our approach is done in two stages, at the regional scale and at the IRIS scale. This double validation is inspired by the work of [25, 51, 52]. It includes:

- The validation of unique sociodemographic indicators (not depending on subgroups of population) such as age or gender at different spatial scales. This simple validation consists solely in checking that the distribution of the modeled data matches that of the census data, for each variable.
- The cross-validation of indicators. This step examines (i) the consistency of the combinations of the different parameters through their occurrence at different spatial scales and (ii) the capacity of the model to reproduce a population at two levels (households and individuals). This step aims to evaluate the model’s ability to reproduce individual’s representative of their environment.

To assess distributions on a regional scale, we have presented histograms of observed and simulated feature distributions by class. To observe the IRIS-scale distribution of sociodemographic features, we plotted scatterplots that represent the percentage of occurrence of the simulated versus observed data, for different parameters at this administrative level. For these tests, the following performance measures were used as indicators of success:

- Coefficient of determination R^2^, which is the square of Pearson’s coefficient. A score close to 1 indicates that our synthetic data closely fit the real data.
- RMSE, the root mean square error, a metric dedicated to quantifying absolute prediction errors.
- MAE which stands for Mean Absolute Error. This measure represents the average error between predicted and observed values.
- SRMSE, which is the standardized root mean square error. SRMSE measures the divergence of an estimated distribution from the true distribution. It is the most widely used measure for assessing the representativeness of marginals and quantifying the closeness of model and observed data sets. It is relevant for discrete attributes, and a zero value means a perfect match between the datasets [53].

### 4.1 Validation of population characteristics

**Fig 3:**
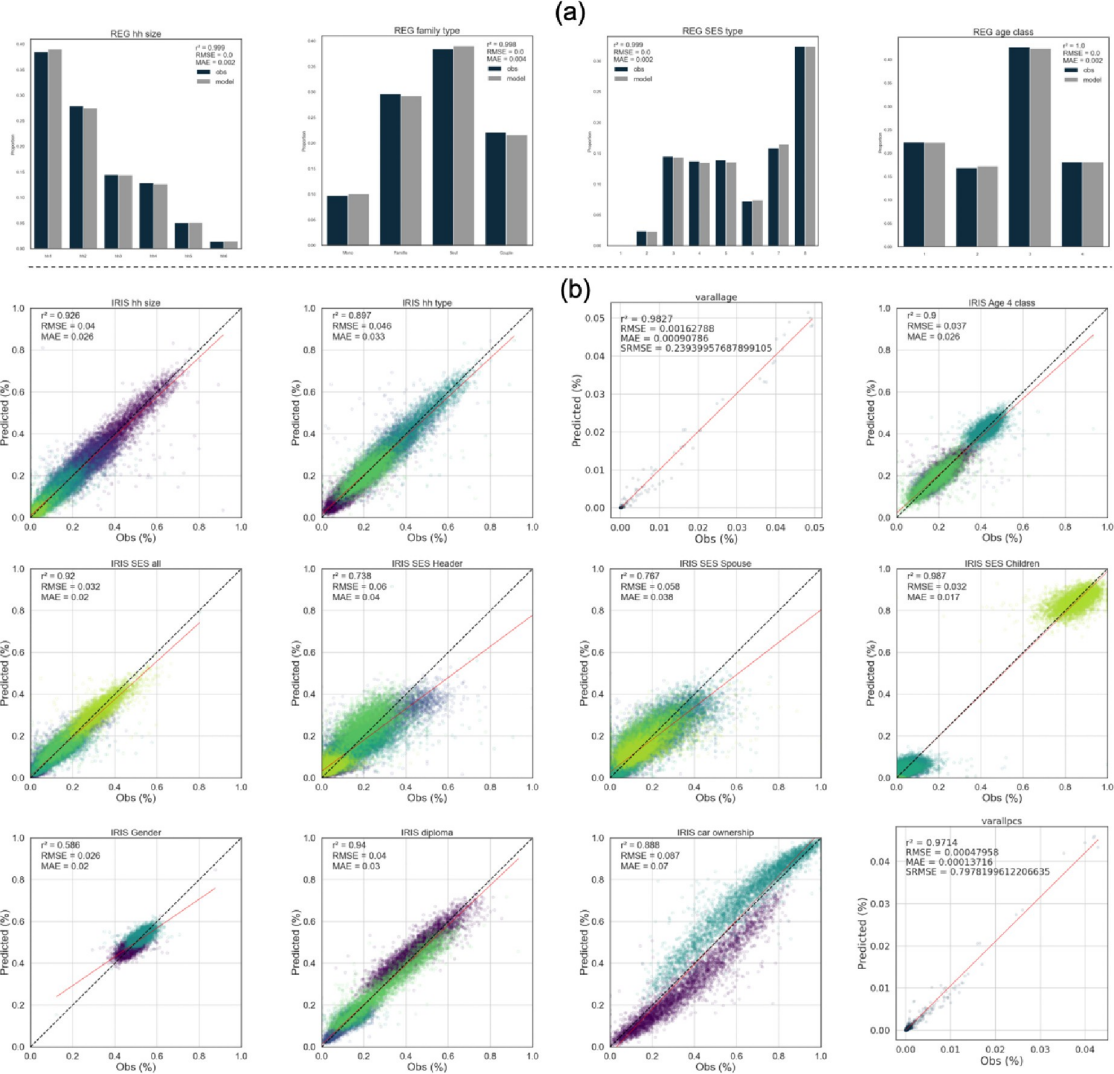
Examples of marginal distributions for the real population of the test database and the samples generated using OLYMPUS-POPGEN for the extended attributes. The bar charts represent distributions at regional level (a) and the scatter plots represent distributions by IRIS (b). The list of variables presented here is not exhaustive. The different colors shown in the scatter plots represent the different attribute classes possible for the corresponding variables.

***Fig 3*** shows a selected subset of 12 graphs illustrating the distributions between OLYMPUS-POPGEN outputs and validation (test) datasets. This figure represents examples of marginal distributions for the real population of the test database and the extended attribute samples generated using the OLYMPUS-POPGEN model. On the top (***Fig 3A***), the bar charts represent the aggregated distributions at the regional level (model in light grey, census in dark bars) while below (***Fig 3B***), the distributions of attributes at sub-municipal scales are represented through scatter plots (OLYMPUS-POPGEN values on the Y axes). The different colors shown in the scatter plot represent the different categories of possible attributes for the corresponding variable. The figure shows both the main structural parameters for housing (size and number of residents per dwelling) and some individual attributes. Overall, the marginal and conditional distributions are well represented in the model.

For unique sociodemographic indicators at the regional level (***Fig 3A***), OLYMPUS-POPGEN provides a very satisfactory representation, with deviations of less than 2% across all classes. The regional population is also very well represented when we look at the details of the 2-, 3- and 4-attribute combinations created by our approach. ***Fig A1* in S1 Appendix** shows, as an example, histograms of the distribution of socio-professional categories derived from the census (in black) and modeled (in red), for different combinations of individual characteristics. The figure shows that the model can reproduce the very wide diversity of SPC distributions across subgroups of individuals, with an occurrence that is remarkably close to that observed. These results give great confidence to the model’s outputs, not only on demographic profiles, but also on social categories of interest for modeling behaviors—notably mobility. Furthermore, the loss of quality for indicators is not observed for RMSEs, which become weaker as the number of constraints increases. Regarding data on overweight and obesity, the evaluation of their distribution in the synthetic population cannot be carried out at the IRIS level (and is therefore not presented in the paper), as this would require cross-referencing with, or even the production of, surveys at this level, which is currently not possible for reasons of individual protection. However, our approach has enabled us to statistically assign the occurrence of overweight and obesity to population subgroups with good confidence, since it is based on the distribution of SPCs that has been validated previously. The validation carried out above therefore allows us to use with greater confidence databases that are not linked to the census but that are strongly correlated with the SPC of individuals.

Still at the regional level, SRMSEs are presented in ***Table A3 in S1 Appendix*** for some main variables. Here, the evaluation is related to the statistical distribution of a given parameter values over the region (shown in the histograms of the previous figure). For the last lines of this table (mentioned as “MVx”), the evaluation of the parameter is related to its distribution among subgroups of individuals having a combination of several Main Variables. Our SRMSE scores range from 0.01 to 2.5 and are thus quite satisfactory, as values up to 10 are generally reported in the state-of-the-art literature [52, 54]. As an example, [55] show R^2^ values close to 0.7 and SRMSEs of around 3 on a cross of 4 parameters for the restitution of a population in the Netherlands, while our SRMSEs do not exceed 0.09 for 4 attributes. These results support the relevance of the OLYMPUS-POPGEN approach, which succeeds in reproducing coherent individuals on a regional scale.

To fully assess the potential of our method, we had to consider increasing complexity in the attribute combinations. Doing this, it was necessary to consider scalability issues. Indeed, it has been shown in the literature that model performance decreases with each addition of a new parameter in the combination evaluated. The contribution of the error related to a variable is thus an increasing function of the number of attributes, and the SRMSE is expected to increase with the complexity of the combination. Such a tendency was observed with Markov Chain methods [30] and [44] also noted that, when using the Gibbs sampling method, an increase in the number of attributes generated can reduce the performance of the model. However, compromise is necessary as increasing the number of attributes synthesized has a positive and significant impact on dimensionality: the number of possibilities is influenced by both the number of attributes and the number of levels within each attribute, which allows the model to better capture the variability of the real population. For our method we have evaluated the contribution to error of adding new parameters with relative changes of the same order of magnitude. We have examined complex cases considering simultaneously up to 9 attributes. The results are shown in the last 5 lines of Table A3 in S1 Appendix (referred to as “MVx” for x Main Variables evaluated). For 6 attributes, the associated error is still comparable with that found in the literature for 4 attributes. In the most complex case, SMRSE reaches 2.56. Such results show that the model can maintain a quite acceptable error rate and underline the effectiveness of our method. This is both encouraging and innovative, as few attempts have been conducted in this direction. Indeed, [44] indicate that, in the literature, most evaluations do not exceed 6 combined attributes.

The demographic and social characteristics of the census at IRIS level shown in ***Fig 3B*** are quite variable, which is consistent with the highly heterogeneous nature of the Paris region demography. And by structure, not all IRIS are covered by the census which makes modelling more challenging. The evaluation of the model is therefore based on its ability to extrapolate learning data to new IRIS. The graphical representation of the measurement-model comparison of these characteristics logically shows greater dispersion. Despite this, the graphs show a high degree of consistency in the model results, with R^2^ values from 0.7 to 0.98 confirming the strength of the correlation between census and predicted data. Secondly, the data well fit the perfect (intercept 0, slope 1) line, in dots. As an exception, the gender distribution has poorer indicators, but this is due to the fact that this characteristic is distributed over a very small range of variabilities. Still, these data remain well distributed along the 1/0 line. Beyond the relevance of the correlation, the R^2^ coefficient and the good alignment of the data also indicate good variability in the properties of the modeled population compared to the real population [23]. The data generated therefore reproduce the real distribution with satisfactory accuracy, but also in all its variability, making it realistic and suitable for risk studies based on individual and collective practices.

### 4.2 Households’ distribution validation

The objectives of our household distribution approach are twofold. Firstly, allocation within dwellings will eventually enable us to characterize atmospheric pollutant concentrations in the immediate environment of dwellings, that is according to their distance from major traffic axes, and thus to assess individual exposure in greater detail. Secondly, it enables us to recreate spatial variability within the IRIS by considering the social profile of individuals, since the distribution of households in dwellings is based on the hypothesis of a positive dependency between price per m^2^ and salary.

The validation of such a distribution at the scale of buildings is not possible due to the lack of data available at fine scale, for data protection reasons (RGPD General Data Protection Regulation). We have therefore plotted the distribution of households, to check the realism of the distribution–i.e. the fact that the urban fabric heterogeneity (collective housing, individual housing) and gradients are correctly represented.

**Fig 4:**
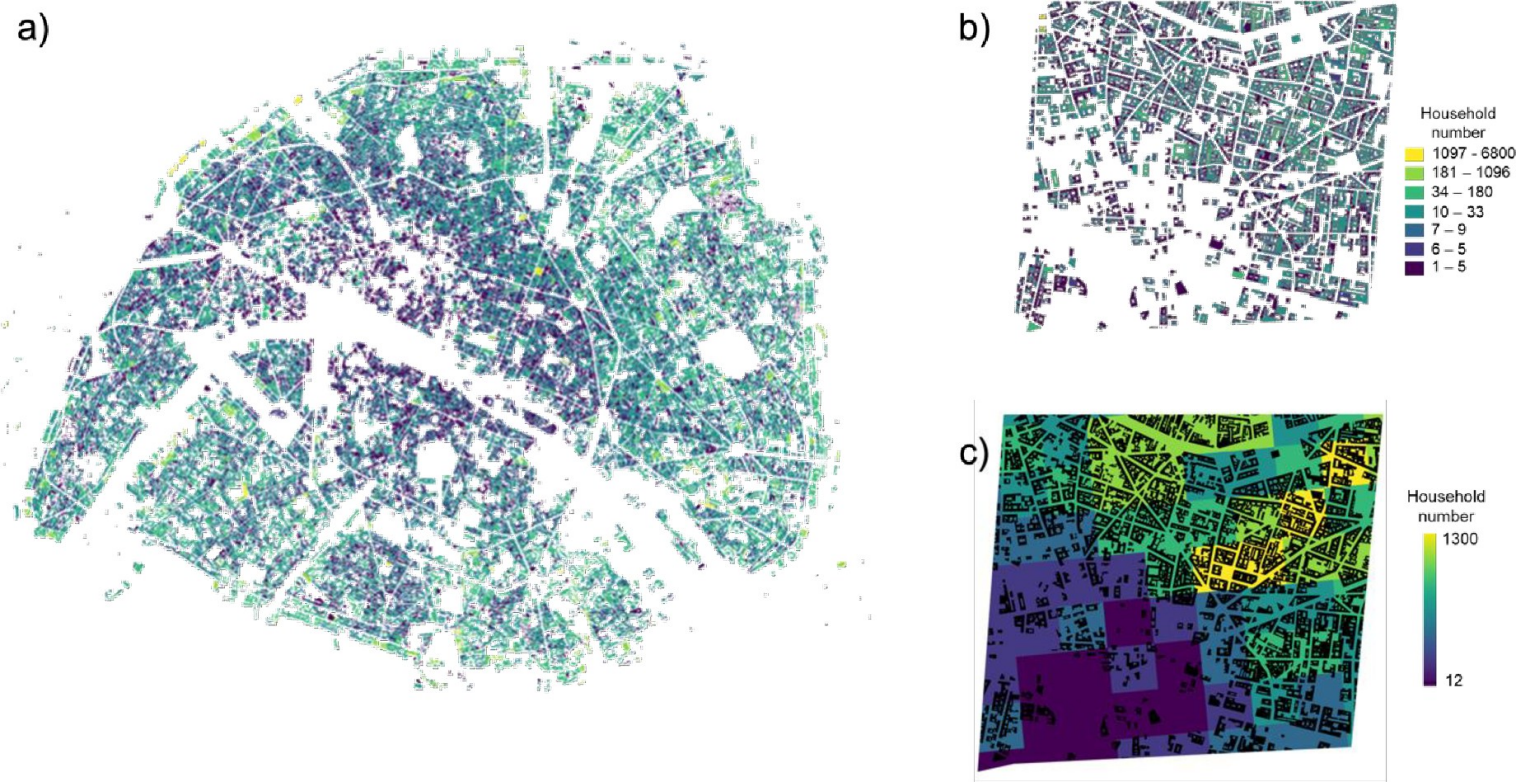
Spatial distribution of data. (a) Spatial distribution of households for the city of Paris. (b) Spatial distribution of households in buildings in the 9th arrondissement of Paris. (c) Spatial distribution of households by square of residence (made using census data, INSEE).

The map of our results is presented in Fig 4 for the city of Paris, focusing on the 9th district of Paris. ***Fig 4(A)*** shows the total number of dwellings per modelled building in Paris. Here we can see that we have managed to reproduce the major expected gradients of household density, with buildings becoming more and more densely populated as we move away from the center of the city, where there are more shops and offices. We zoomed in on the 9^th^ district of Paris and compared our results with census data, for which we have the number of households per 200-meter square. This is shown in ***Fig 4(B)*** for modelled data and ***Fig 4(C)*** for the census. Note here that the spatial unit on which the representation is based is different according to the data source, and it is therefore difficult to assess absolute values. However, we can observe similar trends: in the bottom left-hand corner of ***Fig 4(B)*** we have white areas with relatively sparsely populated buildings, which corresponds to the lowest number of households in ***Fig 4(C)***, that is the purple tiles. In the upper right-hand corner, we have a higher density of buildings and number of households, and if we add them up, we obtain values that are again comparable to those observed in the green-yellow and yellow tiles of ***Fig 4(C)***. In this way, we can consider that our model reproduces the correct trends.

Although the evaluation of our distribution of households in dwellings by income is not feasible due to a lack of refined data, the positive assessment previously conducted on SPC at the regional and IRIS levels enables us to be confident in the results presented here.

### 4.3 Conclusion

OLYMPUS-POPGEN is a code for generating synthetic populations of individuals, households, and dwellings, aiming at a realistic distribution of the socio-demographic characteristics observed in surveys. It includes a data preprocessor, a synthetic population generator and a dwelling locator. We have developed this approach to link with a transport model (whose forcings are conditioned by the differentiated behaviors of individuals in the population) and ultimately with an air quality model, which outputs can be cross-referenced with population respiratory health data to better understand the environmental risk factors exerted on urban populations. The synthetic population was therefore generated to ensure the greatest possible correspondence between the artificial population and the population census data, in an approach where the socio-demographic parameters are linked in a certain hierarchy. The aim of the approach was to generate a population with realistic combinations of attributes through a process in which the agent variables are conditional. To this end, an MCMC method based on Gibbs sampling was implemented, as it appeared particularly suitable to our goals from the literature. However, such an approach had not yet been evaluated on complex combinations of socio-demographic attributes, at several levels (individuals, households) and on a fine scale (IRIS), as we propose in this article.

The model works as follows. First, iterative sampling produces marginal distributions specific to each geographical area. Subsequently, an optimizing approach was implemented to converge the total number of individuals within each zone, while defining household categories in terms of size and typology. The approach then focused on building household agents using conditional probabilities obtained by analyzing survey data. Finally, the distribution of households within buildings and dwellings was carried out based on the corresponding property values.

The advantage of this method is that it does not require the use of rare data or data specific to a particular study region and can be implemented using standard public data. It is easily adaptable to other regions of France and can be extended to other regions of the world, provided that population census data, the French topographical database (BDTOPO) and income data are available. These prerequisites constitute the minimum configuration of data required to ensure a successful application of this approach.

Our methodology, while comprehensive, has certain limitations. The accuracy of the synthetic population is deeply rooted in the quality and reliability of the input datasets. Any biases or inaccuracies present in these foundational datasets can spill over into our synthetic population, potentially distorting the resulting analyses. A particularly complex part of our approach is the allocation of households to specific dwellings, mainly because comprehensive data on fine-scale dwelling occupancy is lacking, as we saw in the comparison of household spatial allocation mappings. This allocation, even though rigorously designed, therefore introduces a degree of uncertainty which could have repercussions in later stages of the analysis, especially in research domains like mobility and environmental exposure, but this uncertainty cannot be assessed.

We applied the approach in the Paris region to model a population of around 12 million individuals, 5.5 million households, in some 5,200 administrative zones. The simulation results show satisfactory reproduction scores for the census population. The simulation results show satisfactory reproduction scores for the census population. The model reproduces the population’s attribute distributions, and attribute-combination distributions, on a regional scale, with a high degree of accuracy. In particular, the scores obtained on combinations of 4 and up to 9 attributes (the latter being rarely, if ever, addressed in the literature) are highly competitive with the approaches and scores reported in the literature. On a fine scale (residential IRIS are homogeneous blocks of 1,000 to 5,000 inhabitants on average), the approach we have implemented also enables us to reproduce with good statistical confidence the spatial distribution of individual properties, their occurrence and their range of variability. For work on mobility and exposure of the population, this makes it possible to generate populations that are representative of the territory, and to exploit the diversity of practices at the origin of environmental risks. As mentioned above, however, the difficulty of validating the assignment of households to buildings and dwellings remains a major limitation in this context. This represents an important way for improvement and gains in robustness for population modelling, but it will be necessary to be able to access databases enabling a link to be made between the specific nature of the dwelling and the type of household residing in it.

As for transposability, although we successfully achieved modelling in the Paris region, translating this approach to other geographical areas may not be straightforward. Variations in socio-demographic nuances, urban configurations and intrinsic quality of data may introduce unpredicted variations in the synthetic outputs for different regions.

This paper completes the existing literature on the issue of synthetic population modelling by working on the question of individual profiles, and by including data exogenous to the usual censuses, such as health-related data, using a spatial distribution of household dwellings. Our synthetic population model offers transformative prospects for several research sectors. In the field of transport, it may assist urban planning by basing studies on both demography-dependent behaviors and socio-economic data. For air quality, it may help predict pollutant emission distribution, as it is highly dependent on mobility practices, and assess the impact of urban life on environmental exposure with possible inequality issues. The model’s versatility also aims to cover other parameters depending on classic socio-demographical variables, as we explored in our approach the feasibility of integrating health data for population subgroups. However, this adds a level of complexity to the population, which performance and uncertainties cannot simply be assessed. Thus, there may be local disparities or inconsistencies between health data and socio-demographic datasets that may not be considered in the synthesized population profiles. New ways of assessing the distribution of variables implemented in the population in a post-processing phase need to be developed.

All in all, OLYMPUS-POPGEN aims to feed decision-support tools for urban management and health and environmental strategies.

Thus, in the future we aim to be able to validate this approach on increasingly complex synthetic populations so that other indicators or cross-reference other databases can be processed and help refine behavioral or risk models. Also, by identifying databases that link household characteristics to specific dwellings, to have feedback on the method accuracy and to be able to refine it.

Our study proposes new angles of approach for synthetic demographic studies, by broadening the panel of data to be considered, reflecting on the constitution of coherent complex profiles, and making household location more comprehensive. It aims to lay the foundations for new, more integrated city-environment-health studies in the future.

## Supporting information

S1 Appendix (DOCX)
