# Supplementary material for: OLYMPUS-POPGEN: A synthetic population generation model to represent urban populations for assessing exposure to air quality
Source: PLoS One. 2024 Mar 8;19(3):e0299383. doi: 10.1371/journal.pone.0299383 (PMC10923402; doi:10.1371/journal.pone.0299383)
Supplement: S1 Appendix — (DOCX) [file pone.0299383.s001.docx]

Table A1: Constraints and sources of datasets for the synthetic population modelling.

|  | **constraints** | **Source datasets** |
| --- | --- | --- |
|  | Age / Gender / Status /Household type / Household size /  SPC / Diploma / Car ownership | Individuals located in the canton-or-city in 2010. Population census - Detailed files - <https://www.insee.fr/fr/statistiques/fichier/2411430/rp2010_indcviza_txt.zip> |
|  | Income | Income structure and distribution indicators in 2010 – Localized household tax income (RFLM) - <https://www.insee.fr/fr/statistiques/fichier/1893299/structure_distrib_revenus_iris_2010.zip> |
|  | Obesity | National epidemiological survey on overweight  and obesity. - <https://presse.inserm.fr/wp-content/uploads/2012/10/obepi_2012.pdf>  <https://liguecontrelobesite.org/actualite/forte-progression-de-lobesite-en-france-en-2020> |

Table A2: Median income by SPC and income coefficients by SPC

| **Socio Professional Categories (SPC)** | **Median annual income in 2014 (r_SPC_)** | **Coefficient (α)** |
| --- | --- | --- |
| Farmers - Business owners | 19290 | 0,92 |
| Managers | 32380 | 1,54 |
| Intermediate prof. | 23810 | 1,13 |
| Employees | 19370 | 0,92 |
| Workers | 18520 | 0,88 |
| Retired | 20880 | 0,99 |
| Inactive | 19510 | 0,93 |
| Other inactive | 14230 | 0,68 |
| General population (r) | 20210 | 1 |

Table A3: Validation parameters for the comparison of simulated and census distributions at the regional level. In the last lines, “MV” indicates that the parameter distribution is evaluated for subgroups of individuals with a specified profile of Main Variables. The first 3 parameters of the profile are the IRIS, the household size and the household type. Then the number of parameters is increased from the precedent line. The left column label stands for “MVx - x^th^ added parameter”. The right column evaluates the change in SRMSE induced by increasing MV.

| *Parameters and*  *constraint level* | *R^2^* | *MAE* | *RMSE* | *SRMSE* | *Relative change* |
| --- | --- | --- | --- | --- | --- |
| *Household size* | *0.9997* | *0.0019* | *0.000* | *0.013803* | *-* |
| *Household type* | *0.9986* | *0.0039* | *0.000* | *0.015681* | *-* |
| *Gender* | *0.9477* | *0.0049* | *0.000* | *0.009751* | *-* |
| *Age* | *0.9536* | *0.0009* | *0.000* | *0.148714* | *-* |
| *Age (4 classes)* | *0.9368* | *0.0253* | *0.026* | *0.104685* | *-* |
| *SPC* | *0.9513* | *0.0167* | *0.020* | *0.165147* | *-* |
| *SPC Head* | *0.8817* | *0.020* | *0.014* | *0.245197* | *-* |
| *SPC Child* | *0.9816* | *0.0231* | *0.037* | *0.295292* | *-* |
| *SPC Alone* | *0.962* | *0.0126* | *0.020* | *0.154390* | *-* |
| *SPC Couple* | *0.9061* | *0.0239* | *0.037* | *0.295686* | *-* |
| *SPC Single parent* | *0.9407* | *0.0229* | *0.036* | *0.290823* | *-* |
| *SPC Family* | *0.9771* | *0.0153* | *0.020* | *0.167783* | *-* |
| *SPC Couple-Head* | *0.9656* | *0.0159* | *0.024* | *0.201912* | *-* |
| *SPC Couple-Spouse* | *0.8063* | *0.0319* | *0.050* | *0.399803* | *-* |
| *SPC Family-Head* | *0.9951* | *0.0056* | *0.000* | *0.054508* | *-* |
| *SPC Family-Spouse* | *0.9654* | *0.0158* | *0.020* | *0.16214* | *-* |
| *SPC Family-Child* | *0.9859* | *0.0209* | *0.033* | *0.267689* | *-* |
| *SPC Single-Parent-Head* | *0.8758* | *0.0262* | *0.036* | *0.290925* | *-* |
| *SPC Single-Parent-Child* | *0.9551* | *0.0317* | *0.051* | *0.404917* | *-* |
| *Diploma* | *0.9345* | *0.0270* | *0.032* | *0.128020* | *-* |
| *Car ownership* | *0.9245* | *0.0481* | *0.048* | *0.096125* | *-* |
| *MV4 - Gender* | *0.9947* | *0.0014* | *0.000* | *0.086451* | *-* |
| *MV5 - Age* | *0.9827* | *0.0009* | *0.000* | *0.230269* | *+166%* |
| *MV6 – Main activity* | *0.9703* | *0.0004* | *0.000* | *0.542738* | *+136%* |
| *MV7 - SPC* | *0.9714* | *0.0001* | *0.000* | *0.731962* | *+35%* |
| *MV8 - Diploma* | *0.9623* | *0.0001* | *0.000* | *1.264875* | *+73%* |
| *MV9 – Car ownership* | *0.8943* | *0.0000* | *0.000* | *2.559091* | *+102%* |


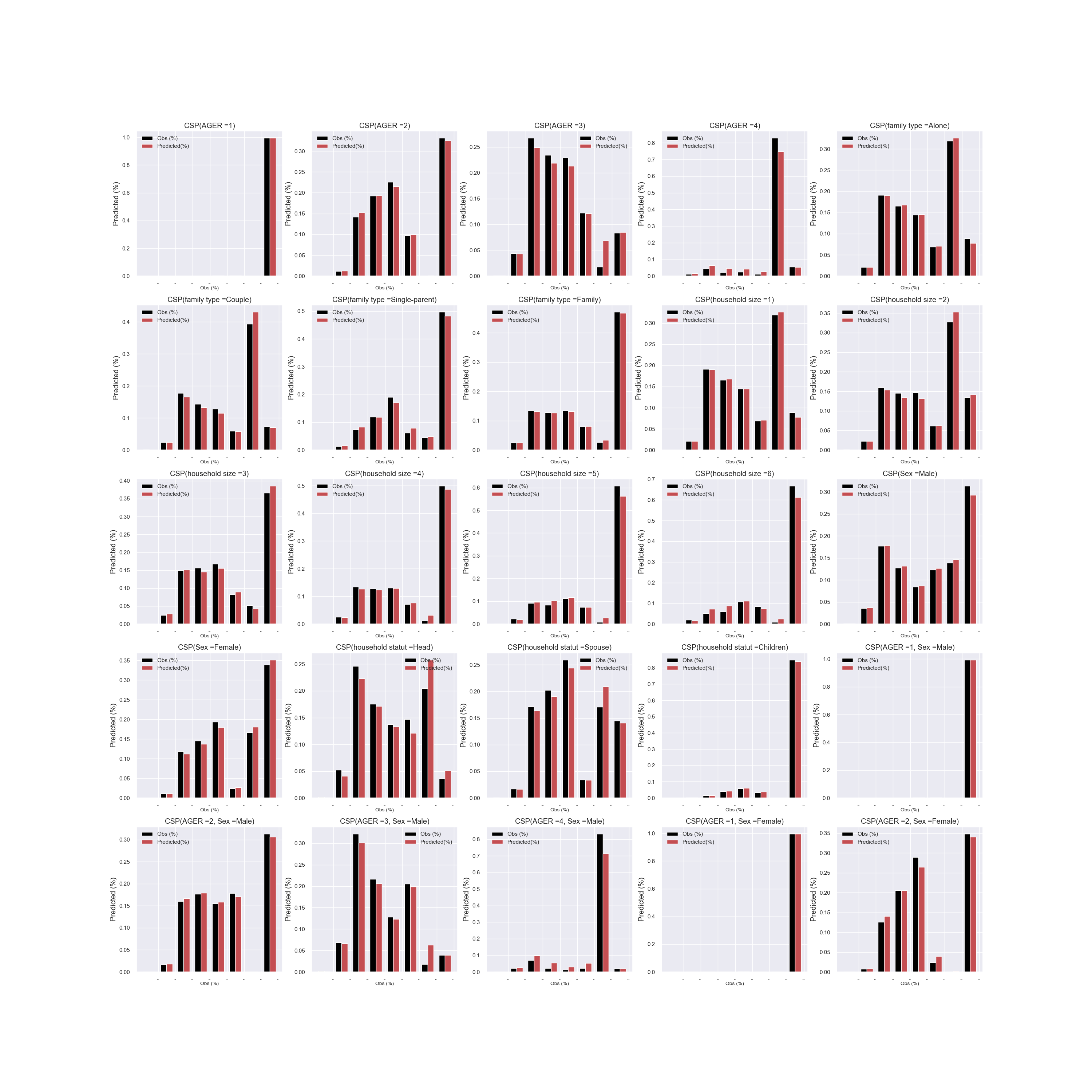


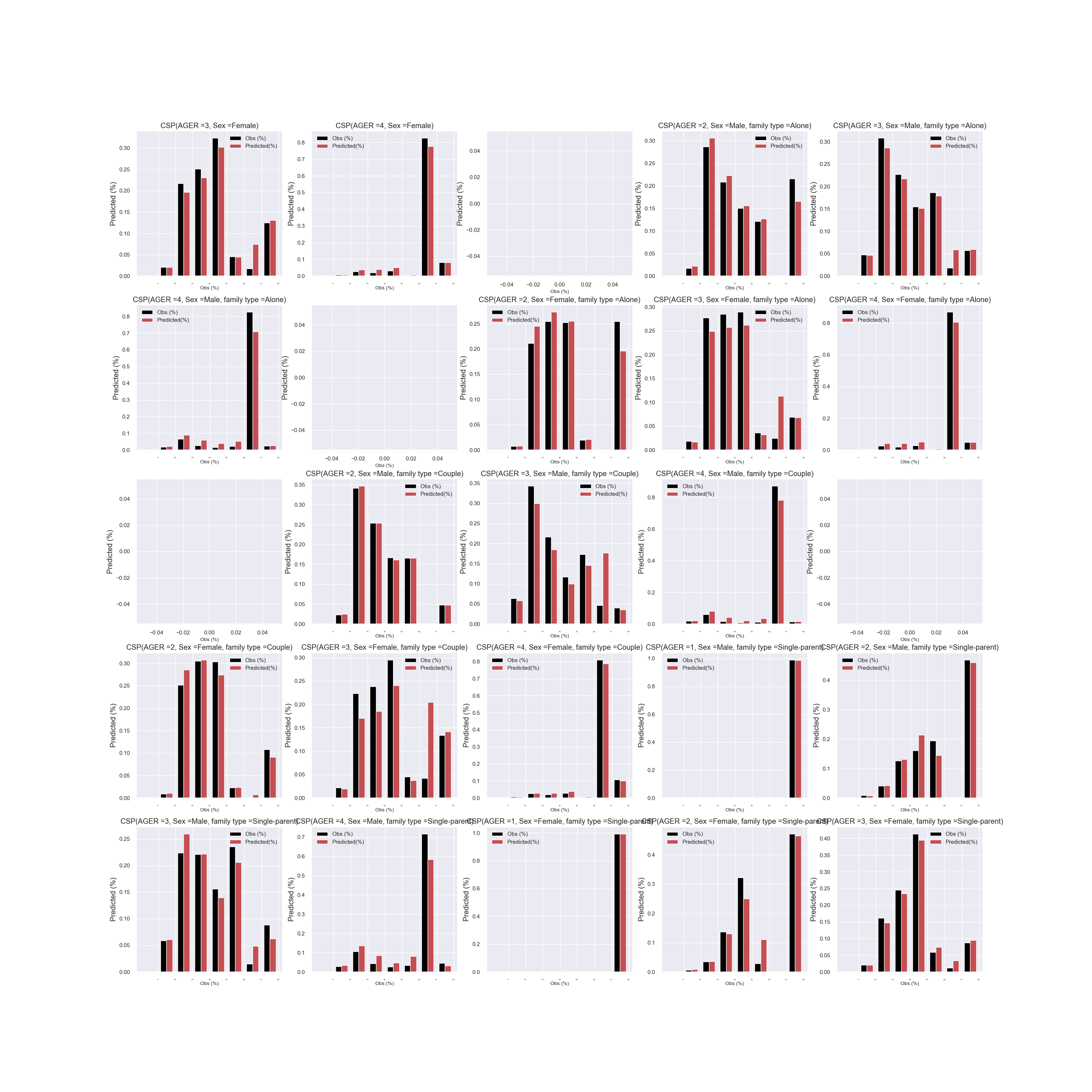
Figure A1: Marginal distributions of socio-professional categories for subgroups of individuals with 1 to 3 demographic attributes, for test (black) and modelled population (red)
